# Supplementary material for: Biosynthesis and bioactivity of anti-inflammatory triterpenoids in Calendula officinalis
Source: Nat Commun. 2025 Jul 28;16:6941. doi: 10.1038/s41467-025-62269-w (PMC12304140; doi:10.1038/s41467-025-62269-w)
Supplement: Supplementary file 2 — Description of Additional Supplementary Files [file 41467_2025_62269_MOESM2_ESM.pdf]

Description of Additional Supplementary Files

Supplementary Data 1: NMR data for faradiol palmitate

Supplementary Data 2: Comparison of experimental and literature assignment of NMR data for faradiol palmitate

Supplementary Data 3: Statistical tests used in Figure 2

Supplementary Data 4: Candidate OSC genes identified in the Calendula officinalis genome

Supplementary Data 5: Coding and translated protein sequences of Calendula officinalis OSC genes

Supplementary Data 6: OSCs used in the phylogenetic analyses in Figure 3

Supplementary Data 7: Statistical tests used in Figure 4E

Supplementary Data 8: Statistical tests used in Figure 5E

Supplementary Data 9: Statistical tests used in Figure 6D and Supplementary Figure 31 Supplementary Data 10: Statistical tests used in Figure 7B

Supplementary Data 11: Plasmids used in this study

Supplementary Data 12: Primers used in this study
